# Magnetogels: Prospects and Main Challenges in Biomedical Applications

**DOI:** 10.3390/pharmaceutics10030145

**Published:** 2018-09-04

**Authors:** Sérgio R. S. Veloso, Paula M. T. Ferreira, J. A. Martins, Paulo J. G. Coutinho, Elisabete M. S. Castanheira

**Affiliations:** 1 Centre of Physics (CFUM), University of Minho, Campus de Gualtar, 4710-057 Braga, Portugal; sergioveloso96@gmail.com (S.R.S.V.); pcoutinho@fisica.uminho.pt (P.J.G.C.); 2 Centre of Chemistry (CQ-UM), University of Minho, Campus de Gualtar, 4710-057 Braga, Portugal; pmf@quimica.uminho.pt (P.M.T.F.); jmartins@quimica.uminho.pt (J.A.M.)

**Keywords:** hydrogels, magnetic nanoparticles, magnetic hyperthermia, drug delivery, cancer therapy, biomedical applications

## Abstract

Drug delivery nanosystems have been thriving in recent years as a promising application in therapeutics, seeking to solve the lack of specificity of conventional chemotherapy targeting and add further features such as enhanced magnetic resonance imaging, biosensing and hyperthermia. The combination of magnetic nanoparticles and hydrogels introduces a new generation of nanosystems, the magnetogels, which combine the advantages of both nanomaterials, apart from showing interesting properties unobtainable when both systems are separated. The presence of magnetic nanoparticles allows the control and targeting of the nanosystem to a specific location by an externally applied magnetic field gradient. Moreover, the application of an alternating magnetic field (AMF) not only allows therapy through hyperthermia, but also enhances drug delivery and chemotherapeutic desired effects, which combined with the hydrogel specificity, confer a high therapeutic efficiency. Therefore, the present review summarizes the magnetogels properties and critically discusses their current and recent biomedical applications, apart from an outlook on future goals and perspectives.

## 1. Introduction

Cancer is still a major leading cause of death worldwide, with lung, colorectal, breast and prostate exhibiting the highest incidence ratio [1,2]. Besides the individual genetical predisposition to develop cancer, such high incidence is also related to risk factors, such as smoking and excess body weight, which may be overcome by the promotion of healthy lifestyles, tobacco control, vaccination, early detection and improved treatments [1,2,3]. Even though medicine progress improved cancer survival rates, such has only been practically verified in high-income countries [3]. Indeed, conventional chemotherapy has been showing good results in patients with early stages of the disease [4]. Yet, patient’s quality of life, target specificity, therapeutic effectiveness and cost have to be readily improved and regarded as a main problem to solve in the next decades, to reduce the adverse effects on healthy cells and extend better treatments to low income countries. Not only cancer therapy but also antibiotics have been a target of intensive research facing more resistant bacteria every year, which will inevitably end in higher susceptibility to untreatable pathogenicities. Such problems require the development of new nanosystems with high performance in therapeutics, which focus on solving the lack of conventional chemotherapy target specificity, improve the treatment of bacterial infections without the use of antibiotics and add further features such as enhanced magnetic resonance imageology, biosensing and hyperthermia.

Since the pioneering work of Wichterle and Lim [5], elastic three-dimensional networks made of water-soluble polymers [6,7], which cross-linking or entanglement retains a huge portion of solvent [8], thus being known as hydrogels, have been thriving in biomedical applications. The core strategy and interest of using hydrogels is mostly centered in taking advantage of the huge diversity of physical and chemical properties passable of being conceived due to the vast variety of usable polymers, producible physical forms and stimulus to activate their formation [6,7,9,10], thereby allowing the development of nanosystems highly adjustable to the needs [11]. Such properties are highly relevant as they allow precise control over drug diffusion and range of drugs capable of being incorporated through the change of networks structure porosity, the hydrogelator affinity to the aqueous environment and density of reticulate agents [6].

Self-assembled hydrogels have acquired a remarkable plethora of applications either in tissue engineering, in vivo imaging or drug delivery, owing to their high portion of water and physical-chemical similarity to the cellular matrix, both in composition and mechanical properties [12,13,14,15,16,17,18,19]. The gelation is achieved through the cooperative effect of different non-covalent intermolecular interactions: hydrogen bonding, van der Waals, electrostatic, hydrophobic and aromatic π-π interactions [7,9,12,13,14,15]. Therefore, the design, preparation and manipulation of the hydrogel must be cautious and rationally conceived since the gelation is affected by pH value, kinetics of pH drop, temperature, ionic force and structure amphiphilicity [7,9,13,14,15,16,17,18,19,20].

On the other hand, magnetic nanoparticles are a growing trend mainly due to the phenomenon of superparamagnetism, as it allows the direct control to a specific location by using a magnetic field gradient [21,22,23,24]. Such phenomenon, which generally happens at sizes smaller than 30 nm, has triggered the research in the synthesis of a huge variety of these nanomaterials made of any transition metal (Fe, Ni, Co, Mn…) and its oxides [21]. Transition metal ferrites are a class of soft-magnetic nanoparticles with great superparamagnetic behavior and other suitable properties for biomedical applications, such as chemical stability and mechanical hardness [22,23,24,25,26]. Within this class of magnetic nanoparticles, manganese ferrite nanoparticles are of interest for biomedical applications, as their magnetic susceptibility is higher than those of other transition metal ferrites [22,24,27,28]. Yet, magnetite (Fe_3_O_4_) and maghemite (γ-Fe_2_O_3_) have been the most used nanoparticles due to their biocompatibility, high magnetization and easy synthesis. Therefore, magnetic nanoparticles have promising properties for biomedical applications, as contrast agents in magnetic resonance imaging, drug delivery and hyperthermia [22,29,30,31].

However, when it comes to in vivo applications, bioaccumulation, bioelimination/excretion, tissue invasion and toxicity have to be taken into account [24,29]. The latter is generally surpassed by using an organic or inorganic shell and the former by using nanoparticles with diameters smaller or comparable to 50 nm, so that it may become possible for nanoparticles to penetrate endothelial fenestrations in capillaries around tumor tissues and having a moderate retention time, which is incompatible with the superparamagnetic behavior [24,29,32].

Hence, taking into consideration the need to develop highly efficient nanosystems and the properties of hydrogels and magnetic nanoparticles, both have been combined to attain magnetogels. Magnetogels ally the properties of both materials and introduce newer ones which are not obtainable when the components are separated, thus improving the effectiveness of both materials mainly in biomedical applications, which will be discussed in the following sections (Table 1). The application of a magnetic field gradient allows the control and targeting of the nanosystem to a specific location. This capability, combined with hyperthermia and enhanced drug delivery promoted by the application of an alternating magnetic field (AMF), and the hydrogel specificity confer a high therapeutic efficiency. In this review, magnetogels properties will be initially discussed, followed by a critical analysis of their current and recent biomedical applications as a new promising nanosystem.

## 2. Properties and Applications of Magnetogels

Upon a stimulus, such as sonication, light irradiation and changing in ionic force or heating, gelation takes place resulting: (I) in the formation of a crystal, due to high ordered aggregates; (II) random aggregation into a precipitate; (III) a metastable state (hydrogel) as a product of optimum intermolecular forces during a homogeneous self-assembly [16]. A reversible supramolecular intertwined fibrillar structure is formed encompassing microdomains of solvent pocket capable of retaining nanoparticles, which may interact with the hydrogelator through non-covalent or covalent bonding, thus modifying network structure [16]. Such a combination can either enhance the pristine materials properties, such as better adsorption or afford further mechanical, electrical, optical and thermal properties [16,43].

A greater control over the mechanical properties is highly important as it allows the tailoring of nanomaterials to mimic the structure of native tissues at the site of implantation and/or the efficient transport and bioaccumulation of growth factors, drugs and cells into a specific location, reducing side effects and enabling higher doses [14,44].

A thorough evaluation of the effect of nanoparticles in the hydrogel matrix mechanical properties was carried out by Bonhome et al. [14], having demonstrated that it strongly changes the storage modulus, rigidity and viscoelastic moduli, where a proportionality with the nanoparticle content is observed. The proposed theoretical explanation is based on the condensation of the polymer in dense phase nuclei, which evolution progressively slows down due to depletion of the supersaturated polymer and reduction of mobility owing to the ongoing polymerization that leads to the nuclei working as polymer fibrils cross-linkers [14]. Furthermore, the authors showed that nanoparticles may function as nucleation sites, working as knots that might be explored as a mean to regulate the porosity, swelling degree and gelation time [14]. In fact, it is demonstrated that, until a nanoparticle critical concentration is attained, the swelling degree increases due to a higher porous structure, while above it the cross-linking effect by the nanoparticles impart less porosity to the network, that consequently leads to a lower swelling degree. However, it should be noticed that an interaction between the nanoparticles and the polymer is required to such effect be observed, as the polymer used by the authors was fibrinogen and could establish ionic interactions with the hydroxide groups of nanoparticles surface.

Indeed, this assumption can be confirmed in many works that studied the incorporation of magnetic nanoparticles in polymer hydrogel matrixes to improve drug delivery efficacy but have many different outcomes. For example, Hamidian et al. [45] incorporated magnetite nanoparticles into a starch-based grafted with poly(ethylene phthalate) hydrogel, which enhanced a higher swelling degree but worsens the drug loading. On the contrary, Mahdavinia et al. [46] developed k-carrageenan and chitosan-based magnetogels with magnetite nanoparticles that exhibit increased methotrexate loading efficiency with higher magnetite content but lesser swelling degree. Therefore, the effect of nanoparticles on the hydrogel matrix should always be evaluated to attain the best outcome for the desired application.

Apart from the presence of magnetic nanoparticles allowing their detection and control through a magnetic field gradient, it also stimulates cell adhesion, proliferation, differentiation and enables the visualization and in vivo follow-up though magnetic resonance imaging [14]. A magnetogel of alginate-*g*-heparin with magnetite nanoparticles was developed by Kim et al. [33], the structure of which was modified by changing the components concentration and by the application of a magnetic field [33]. The authors demonstrated that the application of a magnetic field allowed control over the release of transforming growth factor beta 1 (TGF-β1) from the magnetogel network enhancing the differentiation of ATDC5 chondrogenic cell line [33]. Hereby, the control over diffusion through an alternating magnetic field reveals the superior efficiency of the magnetogels, where the drug diffusion is enhanced by the thermally induced network collapse and/or vibrational fiber disruption, while hydrogels rely only on the molecular diffusion and hydrogel degradation.

Eventually, the outstanding properties of magnetogels also led to research in industrial applications, such as the development of improved methods of depollution due to the easiness of removal by a magnetic field, the large number of reusable cycles and the huge variety of removable contaminants. For example, Kim et al. [47] developed a potassium copper hexacyanoferrate-immobilized magnetogel with magnetite nanoparticles coated with citric acid to allow rapid recovery through an applied magnetic field and improve KCuHCF dispersion, which may enhance Cs^+^ adsorption. The system showed high efficiency in a broad pH range and in the presence of cations that directly compete with Cs^+^, having a capacity of 82.8 mg/g of magnetogel [47]. Nevertheless, besides attaining highly efficient magnetogels for the removal of nuclear residues [48], it is necessary to design low-cost techniques and materials for the removal of dyes, as they are hardly degraded by microorganisms, light or chemical treatments. This issue, combined with the low efficiency and high cost of the current methods, puts the future of the ecosystem in danger [49]. An example of such materials was achieved by Mittal et al. [49], who developed a gum xanthan-grafted-polyacrylic acid based magnetogel containing Fe_3_O_4_ nanoparticles, which showed a capacity of 642 mg of methyl violet per gram of magnetogel. Comparatively to the hydrogel, the system was prone to better performance owing to the higher surface area and exposure of more binding sites induced by the nanoparticles, thus being a promising material for removal of cationic dyes [49].

**Figure 1:**
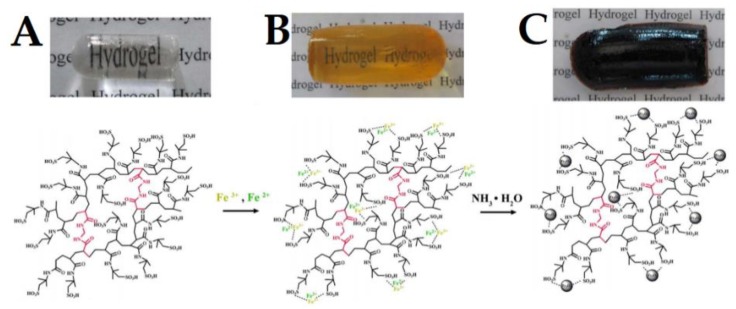
Schematic representation of the PAMPS based magnetogel preparation, where (**A**) the hydrogel is immersed in a Fe (II) and Fe (III) ion-containing aqueous solution which (**B**) are absorbed by the hydrogel network. The nanoparticles are then formed by co-precipitation in an ammonia aqueous solution that (**C**) results in the magnetogel. Adapted from [57] with permission from John Wiley and Sons, 2015.

From the given examples, the reader can already notice that Fe_3_O_4_ nanoparticles are widely used in magnetogels. Indeed, the most commonly used magnetic nanoparticles have been magnetite (Fe_3_O_4_) and maghemite (γ-Fe_2_O_3_) [50,51]. Such fascination with these types of nanoparticles can be explained by their magnetic properties, corresponding to the major requirements of biomedical applications, such as low production cost, good physical and chemical stability, biocompatibility and environmental safety, thus being widely used in target drug delivery, MRI, magnetic hyperthermia and biosensing [52]. However, it has been reported that iron oxide nanoparticles show peroxidase-like activity, thereby producing reactive oxygen species that may cause severe damage to cell components and induce inflammatory responses [53,54,55]. In addition, they tend to accumulate in organs with a high concentration of macrophages and thus may not be advisable for long-term applications such as MRI, taking into consideration that they might disturb the immune function of the reticuloendothelial system [53]. Therefore, the use of iron oxide nanoparticles requires improved coating, either polymeric or inorganic, to reduce these effects, which is expected to be more frequent in future reports, as the techniques of production become cheaper and simpler. Nevertheless, the peroxidase-like activity can be explored for the development of biosensors. For example, Song et al. [56] developed a magnetogel based on cationic surfactants C_n_TAFB(C) (CH_3_(CH_2_)_n−2_CH_2_N(CH_3_)_3_[FeCl_4_] and CH_3_(CH_2_)_n−2_CH_2_N(CH_3_)_3_[FeBrCl_3_], with n = 12, 14, 16) and the chiral amphiphile sodium cholate containing 7.2 nm Fe_3_O_4_ nanoparticles. This system was capable of detecting glucose based on the oxidation of 3,3′,5,5′-tetramethylbenzidine (TMB) in the presence of H_2_O_2_ that results from glucose oxidation by glucose oxidase, producing a blue color with a maximum absorbance at 652 nm and attaining a detection limit of glucose of 38.5 μmol/L. In another approach, Sang et al. [57] developed poly(2-acrylamido-2-methyl-1-propanesulfonic acid) (PAMPS) based magnetogel containing Fe_3_O_4_ and evaluated the effect of cross-linking concentration on the peroxidase-like activity (Figure 1). The system showed the best catalytic activity for low cross-linking concentrations, which the authors suggest being associated to the enhancement of system affinity towards the substrate and due to the good dispersion of Fe_3_O_4_, having attained a detection limit of 1.5 × 10^−6^ mol L^−1^ with a linear detection range of 1.5–9.8 × 10^−6^ mol L^−1^ [57]. The reader is referred to other articles if has an interest in the exploration of catalysis with magnetogels [58,59,60,61].

Apart from all the properties and advantages, just like hydrogels, magnetogels can be designed and fabricated into different types such as spheroid-like structures known as microgels, nanofibrous networks and mesh-like nanocomposites, thus granting more control over the nanosystem, as the optimum structure can be adapted to the desired application [50,62,63,64,65,66,67,68]. For example, an interesting concept to develop microgels is the addition of a silica shell to a magnetic nanoparticle core, which is further grafted with the hydrogelator polymer of interest. This concept was shown by Hinrichs et al. [62] where silica-coated magnetite or hematite nanoparticles were grafted with *N*-isopropylacrylamide (NIPAM) by a free radical emulsion polymerization method, which was further cross-linked using *N*,*N*-methylenebisacrylamide (BIS) and grafted with amine groups at the nanoparticle surface by addition of acrylic acid (AA). Macrogels can then be attained by reaction of amine groups with the aldehyde moiety of glutaraldehyde (GA) [62]. A common method for the development of magnetogels is the mixture of nanoparticles and a hydrogelator solution followed by its gelation that produces a mesh-like composite, which sometimes might require the nanoparticle surface functionalization to grant it stabilization. For example, Tóth et al. [63] developed magnetogels based on hyaluronate (HyA) and magnetite that had to be functionalized due to the flocculation of magnetite nanoparticles by HyA molecules with the formation of aggregates. This effect was prevented by using a natural and biocompatible polymer, chondroitin-sulfate-A, which allows the production of an interesting nanosystem for biomedical applications, such as the treatment of osteoarthritis [63]. Alternatively, this mesh-like composites can be attained by in situ synthesis of magnetic nanoparticles, i.e., the nanoparticles are synthesized directly in the hydrogel matrix [65]. Another common magnetogels design has been the bead-based nanostructures, which are of interest in a wide variety of applications and the general strategy involves the ionic cross-linking of sodium alginate in which can be integrated another polymer to manipulate its properties [66]. A recent design has been the use of supramolecular hydrogelators that can self-assemble in tubular structures or fibers, attaining nanofibrous networks which can also be explored for the development of magnetogels. As an example, Das et al. [68] developed a magnetogel based on diphenylalanine co-assembled with polydopamine spheres coated with magnetite nanoparticles. Hereby considering the morphology, a microgel would be more appropriate for intravenous injection, while the macrogel would be desirable for dermal application, due to its bulk volume. However, when it comes to cancer therapy in deep tissues, it should be noticed that microgels will have a similar behavior to liposomes, in opposite to macrogels that would require intramuscular injection for solid tumor therapy. Thus, besides the targeting of magnetogels through an applied magnetic field gradient, the nanosystem can also accumulate in the tumor region, due to the enhanced permeability and retention (EPR) effect around tumor vasculature, which can be further allied with active targeting by a targeting moiety such as arginine-glycine-aspartate (RGD) sequence or other specific targeting molecule, to increase therapeutic specificity and reduce potential side effects [52].

**Figure 2:**
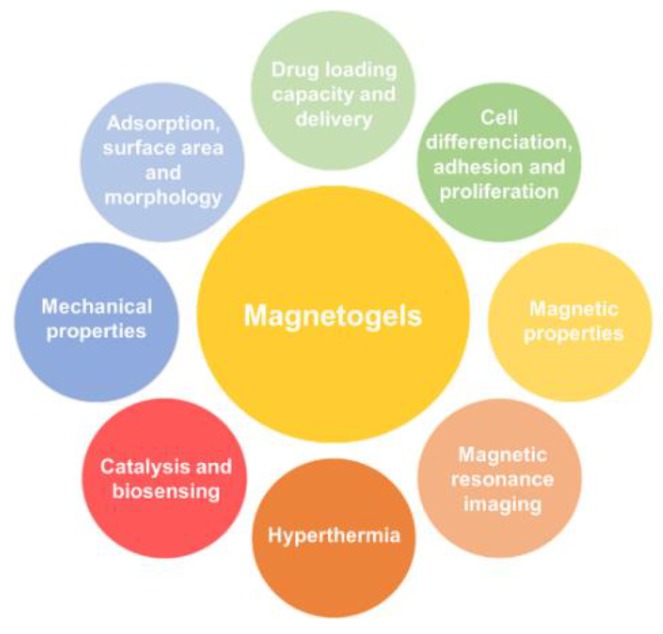
Introduced, tuneable and controllable properties and applications of magnetogels.

Considering all the different advantages associated to the introduction of further tunable and controllable properties previously discussed (Figure 2), magnetogels come up as highly potential candidates for theranostics, being one of the current main biomedical challenges [69,70]. The importance of theranostics is associated to the possibility of combining a non-invasive detection of biomarkers at an early stage of a given disease with the choice, adoption and administration of medical personalized treatments that take into account the genotype and the phenotypic characteristics of the patient [70]. Therefore, the possibility of incorporating an immense variety of chemical and structural functionalities in the same architecture might allow a precise, sensible and accurate diagnostic accompanied by the treatment of heterogeneous pathogenicities, such as cancer [70].

## 3. Hyperthermia, Drug Delivery and Cancer Therapy

Unlike the bulk ferro- and ferrimagnetic materials that generate heat through hysteresis losses, the nanoparticles superparamagnetic behavior induces the conversion of magnetic energy into thermal energy by means of different mechanisms when the material is subjected to an alternating field, showing no remanence magnetization upon magnetic field removal [71,72]. Such mechanisms can be either a Néel or Brownian relaxation, the dominant relaxation process being dependent on the size and the particular magnetic material.

The Néel relaxation becomes the prevailing process when the nanoparticles are small enough to the point it only requires a little bit of energy to induce rotation of the magnetic moment, being highly influenced by the size of the nanoparticle. In these conditions, the relaxation time is given by the equation [71]:

(1) $$\tau_{N}=\tau_{0}e^{\frac{KV,}{k_{B}T}}$$

where $\tau_{0}$ is the characteristic time of the material (10^−9^–10^−12^ s), *K* is the anisotroFpy constant, *V* the particle volume, $k_{B}$ the Boltzmann constant and *T* the temperature.

**Figure 3:**
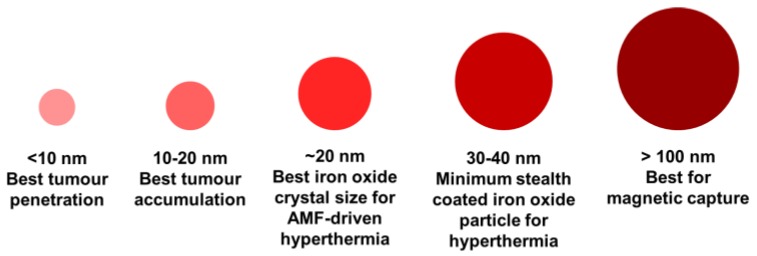
Preferential application of iron oxide magnetic nanoparticles according to their size.

The other side of the coin is the Brownian relaxation, which is affected by medium viscosity, where the relaxation time is given by the following equation [71]:

(2) $$\tau_{B}=\frac{3\eta V_{H},}{k_{B}T}$$

where *η* is the medium viscosity, *V_H_* the particle hydrodynamic volume, *k_B_* the Boltzmann constant and *T* the temperature. The application of a magnetic field faster than the nanoparticles relaxation time will induce heat generation due to the delayed relaxation of the magnetic moments [71]. Therefore, as the temperature generated by the nanoparticle will produce changes in the medium viscosity, which affects the relaxation time and the particle movement, it is preferable the use of nanoparticles with Néel relaxation to avert free particle rotation due to viscosity fluctuation. Nevertheless, these relaxation mechanisms are size and material dependent and thus, this flexibility allows the development of nanoparticles specifically designed for the desired application [70] (Figure 3).

Hence, the concept of magnetic hyperthermia appears where the tissues are exposed to high temperatures (42–45 °C), either to kill tumor cells by apoptosis or to prompt higher susceptibility to radiation and antitumor drugs [31,71]. In this temperature range, enough thermic energy is provided to promote denaturation of cytoplasmic, membrane and nuclear proteins. Such denaturation of proteins required for the synthesis of DNA might induce higher susceptibility of cancer cells to heat, considering that a malfunction mitotic phase will hinder the cellular division [71]. Even though higher temperatures may seem beneficial by inducing apoptosis (around 46 °C) or cell necrosis, such temperatures can also affect healthy cells and so a compromise above 42 °C and below 46 °C is required. Above 42 °C, the blood flux is slowed down in the tumor region, together with a lower oxygen and nutrients concentration, low pH and random vascularity. Consequently, the heat dissipation is hampered and thereupon tumor cells become more sensitive to hyperthermia therapies [23,71].

On the other hand, the application of temperatures below 42 °C will enhance blood flux and oxygenation in tumor tissues that may contribute for a higher drug accumulation, intracellular assimilation and DNA damaging, thus establishing a synergy with chemotherapy [23,71]. Indeed, hyperthermia has become a suitable adjuvant therapeutic technique for combination with chemotherapy, due to its influence over the antineoplastic drugs cytotoxic effect and perfusion facilitation, but also due to the enhancement of local oxygenation that might favor radiotherapy [31].

The multiple magnetic hyperthermia application is advantageous, still, the low nanoparticle retention time does not allow the generation of sufficient heat needed for long-term repetitions, requiring additional injections [31]. Hence, magnetogels may resolve such problem, as the hydrogel matrix grants a long-term retention of the nanoparticles in the application site and reduces tissue invasion by requiring a lesser number of injections.

In the field of hyperthermia, thermoresponsive polymers have been of great interest owing to their phase transition from hydrophilic to hydrophobic upon a change in temperature [50]. Such materials can be either positive (volume increase) or negative responsive (volume decrease) when the temperature increases. For example, poly(*N*-isopropylacrylamide) (PNIPAAm) based hydrogels undergo a phase transition from a swollen to a collapsed state around their lower critical solution temperature (LCST) of 34.6 °C, which can be used to enhance drug delivery [73]. Moreover, PNIPAAm is a biocompatible synthetic polymer which cross-linking concentration can be adjusted to tune the phase transition temperature [73,74]. A study on the use of this polymer in hyperthermia was carried out by Tabatabaei et al. [74] who developed a magnetogel containing iron oxide nanoparticles with a 40 °C phase transition temperature. The authors showed that, upon application of an AC magnetic field of 4 kA/m at 160 kHz, the magnetogel shrunk with a 25% volume reduction, apart from being possible to propel the system by using magnetic gradients of 400 mT/m inside a clinical MRI scanner [74]. Meenach and co-workers [34] also developed a magnetogel based on synthetic polymers, the poly(ethylene glycol) methyl ether methacrylate (PEGMMA) and dimethacrylate (PEGDMA) containing iron oxide nanoparticles. The authors showed that besides the hydrogel matrix improving cell viability compared to only nanoparticles, the system was capable of being heated to the hyperthermic temperature range (41–44 °C) or thermoablative temperature range (61–64 °C) by controlling the magnetic field strength or the iron oxide content [34]. The nanosystem selectively killed M059K glioblastoma cells in vitro by exposure to an AC magnetic field of 25 kA/m at 297 kHz, thus being a promising system for metastasis prevention after surgical tumor resection and for in situ formation in inaccessible locations [34].

**Figure 4:**
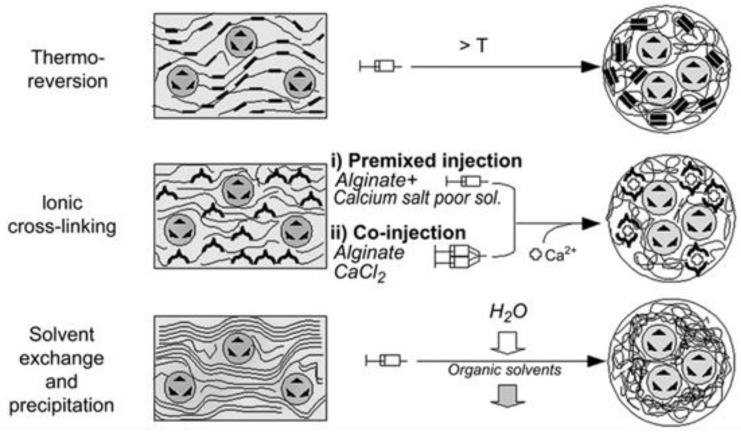
Schematic representation of three possible injection mechanisms for in situ hydrogelation of magnetogels. Reprinted from [75] with permission from Elsevier, 2010.

An interesting work was carried out by Renard et al. [75] that evaluated in situ magnetogel formation with iron oxide nanoparticles embedded in silica microparticles (beads) to favor syringeability of the injectable formulation, in a high concentration to attain large heating capacities, apart from showing different methods for implant in situ formation (Figure 4). The hydrogelation in situ may be beneficial by avoiding potential harmful nanoparticle migration, which combined with hyperthermia approaches will be highly advantageous for solid tumor treatment [75]. For hydrogelators, chitosan and poloxamer 407 were used as models of thermoresponsive hydrogels, with a temperature for sol-gel transition in the interval 33–36 °C, while water soluble sodium alginate with calcium as ionic cross-linker was used to evaluate internal and external hydrogelation as the best approach [75]. The authors demonstrated that the thermoresponsive magnetogels containing 20% *w*/*v* of magnetic microparticles formed an unstable implant due to the high particle content compromising the sol-gel transition and/or the mechanical strength [75]. On the other hand, alginate magnetogel containing 10% *w*/*v* nanoparticles and externally hydrogelated formed a strong implant in the tumor periphery, while internal hydrogelation failed in situ [75]. Thus, the authors suggest that for attaining control over the magnetogel distribution into the tumor center and periphery, a balance between a high viscosity and rapidity of hydrogelation is required [75].

Indeed, this has been the way forward in the last decade, with continuous research on materials with the appropriate mechanical characteristics and microstructure to improve cancer therapy and drug delivery [31,76,77,78,79,80]. In addition, even if synthetic polymers may be the most easily manipulated to acquire the desired mechanical and functional properties, they have the severe problem of low biodegradability, due to the high chemical stability under physiological conditions. Thus, biodegradability is another factor to consider, which can be accessed by using natural hydrogelators or synthetic that are either chemically designed with degradable functionalities or employed in a combination approach [77].

Hyperthermia has been constantly adapted to be fully applied as a stand-alone therapy, either by using a high concentration of nanoparticles or with a high magnetic susceptibility. An example of that is the solution proposed by Vilas-Boas and co-workers [81], where the combination of unfunctionalized nanoparticles with CXCR4-targeted nanoparticles granted a complete loss of cell viability after 72 h, thus surpassing the practical limit that didn’t let the achievement of a 100% lethal therapy. Such studies are highly relevant, as even if hyperthermia as a stand-alone therapy may be impractical to the point that it may require the patient to be submitted too much time to an AMF, the development of methods and strategies that improve the heating performance will be beneficial for the hyperthermia-chemotherapy synergy under an applied magnetic field.

**Figure 5:**
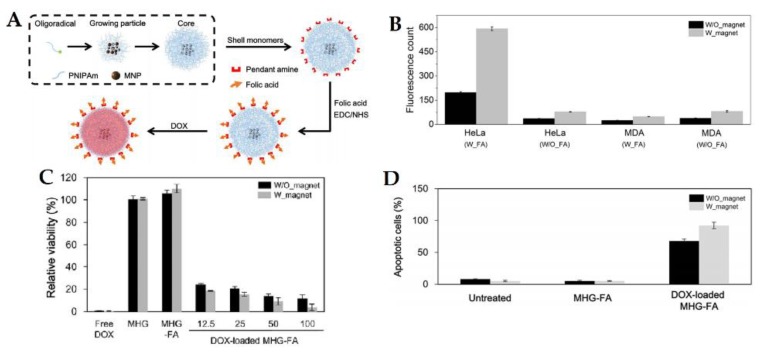
(**A**) Schematic representation of the magnetogel preparation by precipitation polymerization with conjugation of folic acid via EDC/NHS (1-Ethyl-3-(3-dimethylaminopropyl)carbodiimide/N-Hydroxysuccinimide) chemistry and loaded with doxorubicin. (**B**) Quantitative cellular uptake of magnetogels by HeLa and MDA-MB-231 cells with and without and externally applied magnetic field and with or without folic acid. (**C**) Relative viability of HeLa cells treated with free doxorubicin (DOX) (100 μg/mL), magnetogel (MHG) (100 μg/mL), magnetogel with folic acid (MHG-FA) (100 μg/mL) and DOX-loaded MHG-FAs at various concentrations (12.5, 25, 50 and 100 μg/mL), treated for 30 min with or without externally applied magnetic field and incubated for 96 h. (**D**) Apoptosis percentage of HeLa cells treated with 100 μg/mL MHG-FA or DOX-loaded MHG-FA for 30 min and further incubated for 48h. Adapted from Kim et al. [44], Nature Publishing Group, 2017 (available under Open Access).

In a work by Gupta et al. [82], a poly(vinyl alcohol-*g*-2-hydroxymethyl acrylate) (PVA-*g*-PHEMA) based magnetogel was developed containing iron oxide nanoparticles. The system can attain a 55 emu/g saturation magnetization, stability of drug loaded at pH 7.4, enhancement of drug release proportional to the intensity of the applied field and antibacterial activity when loaded with ciprofloxacin, thus revealing promising properties for drug delivery [82]. In another example of magnetic field induced drug release, a salecan-*g*-poly(crotonic acid-co-*N*-(hydroxymethyl acrylamide)) magnetogel containing core/shell nanoparticles of Fe_3_O_4_/SiO_2_ was evaluated as a drug nanocarrier, showing almost no cytotoxicity to A549 and COS-7 cells [83]. The maintained doxorubicin release killed about 73.9% of A549 cells at a concentration of 12 μg/mL, with the potentiality of higher efficiency, considering that a release of 89.5% at pH 4.0 under a magnetic field of 1800 G was achieved [83]. In a similar work, the same authors developed a biocompatible salecan-*g*-poly(vinylacetic acid-co-2-hydroxyethylacrylate) [poly(VA-co-HEA)] copolymer based magnetogel containing Fe_3_O_4_@agarose nanoparticles with a maximum magnetization of 19.8 emu/g, apart from a sustained and pH-triggered release of doxorubicin (DOX) [35]. The application of a 2200 G magnetic field at pH 4.5 accelerated the drug release, with a maximum of 91.3% after 64 h [35]. In another example, Kim et al. [44] developed a magnetogel based on temperature-responsive poly(*N*-isopropylacrylamide), magnetite nanoparticles and folic acid as a specific targeting ligand for cervical cancer cell line (HeLa), thus enhancing intracellular uptake by combining magnetic attraction and receptor-mediator endocytosis (Figure 5). The authors demonstrated that synergy was achieved, further enhancing intracellular uptake, efficient cytotoxicity and apoptotic activity of HeLa cells [44].

In an interesting work by Xie et al. [39], a biocompatible magnetogel based on chitosan crosslinked with telechelic difunctional poly(ethylene glycol) was developed containing superparamagnetic iron oxide nanoparticles and the synergetic behavior of doxorubicin (DOX) and docetaxel (DTX) in a triple negative breast cancer cell line was assessed. The nanosystem showed remarkable results, as the combination of both chemotherapeutic drugs improved the release profiles of each other and the application of an AMF of 19.99 kA/m at 282 kHz capable of inducing a rise of 27.6 °C within 300 s, speeded up the release of docetaxel [39]. In addition, in vitro assays resulted in a cell viability lower than 19.6% when DOX and DTX were combined and an enhancement to 5.6% under the application of an AMF [39]. The in vivo assays also showed the synergetic behavior of hyperthermia and dual-drug release, by improving antitumor activity when mice were subjected to a 10 min AMF for 4 days [39].

Indeed, such work is a remarkable achievement in the field of drug delivery by magnetogels, considering that improved efficiency of tumor treatment is always desirable, mainly the prevention of local recurrence or metastasis immediately after tumor debulking surgery that can be accessed by filling the cavity with an implant capable of controlled drug release [39]. Such purpose cannot rely only on single drug administration considering that the high heterogenicity of tumor tissues and differences in cancer progression may end up in the failure of the desired effect. Thus, an alternative strategy is the synergetic combination of chemotherapeutic drugs with different cytotoxic pathways and toxicity profiles, which might overcome such problem and further enhance the therapeutic efficiency of a single injection, accompanied by a reduction of the required drug dosage as was demonstrated by the previous work [39]. Therefore, this strategy should be taken as a major objective in future developments in the field of drug delivery.

Although doxorubicin is widely used to study drug delivery with magnetogels and other nanosystems due to its high efficiency in many cancer types, it must be considered that its toxicity to cardiac tissues, promoting hypotension and transient electrocardiographic abnormalities, is a drawback to its full potential as a chemotherapeutic agent. Considering this problem, there have appeared some interesting works using natural compounds to reduce doxorubicin side effects [36,84] For example, Namdari et al. [36] developed a magnetogel based on *N*-isopropylacrylamide copolymerized with methacrylic acid (NIPAAM-co-MAA) containing Fe_3_O_4_ nanoparticles and evaluated curcumin effect on the reduction of cardiac toxicity induced by doxorubicin. Doxorubicin can induce free radical oxygen species either by synthesis of a semiquinone free radical that produces a superoxide radical or the formation of an iron-doxorubicin complex capable of reducing oxygen to hydrogen peroxide and other active species, thus being harmful to lipids and peptides in cell membranes. The authors showed that an increase in curcumin-loaded magnetogel resulted in the reduced expression of heart failure and cardiac hypertrophy markers β-MHC (beta major histocompatibility complex), ANP (atrial natriuretic peptide) and BNP (B type natriuretic peptide) [78]. In another example, Cheragi et al. [84] evaluated the cardioprotective effect of *N*,α-L-rhamnopyranosyl vincosamide (VR) encapsulated into a NIPAAM-co-MAA based magnetogel. The authors showed that the application of VR-loaded magnetogel in rats reduced the level of myocardial malondialdehyde (MDA) comparatively to the standalone VR treatment, which is a biomarker associated to the oxidative stress [84]. This result indicates a blocking effect of Dox-mediated lipid peroxidation, which combined with the reduction of the β-MHC, ANP and BNP markers, comparatively to the standalone therapy demonstrates the nanosystem potentiality for a future doxorubicin combined therapy.

**Figure 6:**
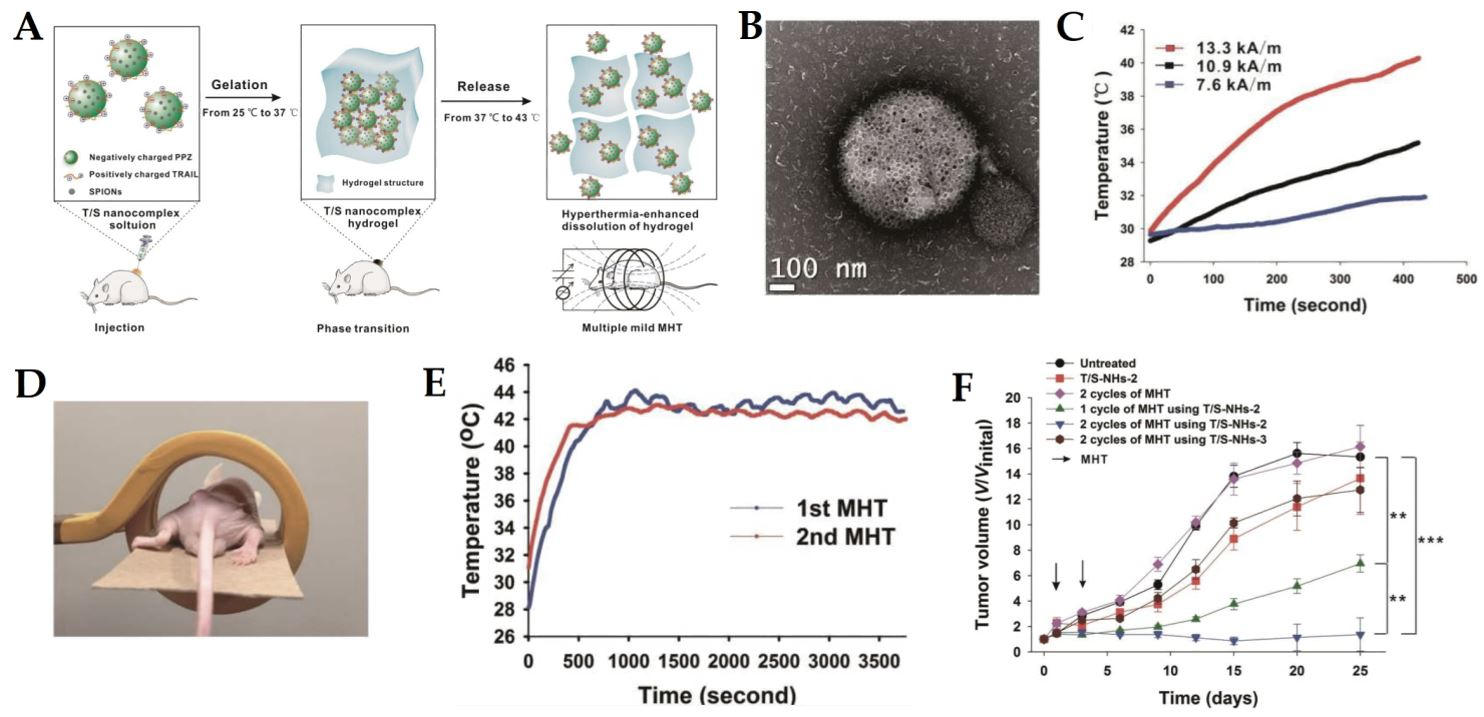
(**A**) Schematic diagram of the thermosensitive magnetogel developed by Zhang et al. based on positively charged tumor necrosis factor-related apoptosis-inducing ligand (TRAIL) and hydrophobic superparamagnetic iron oxide nanoparticles, complexed with negatively charged poly(organophosphazene) polymers, which TRAIL-SPION nanocomplex release from hydrogel is enhanced at 43 °C under multiple MHT. (**B**) TEM representation of TRAIL/SPION nanocomplex. (**C**) Temperature versus time graphs of TRAIL-SPION nanocomplex at 0.5 mg Fe/m over different alternating magnetic field amplitudes and a constant frequency of 366 kHZ. (**D**) Schematic illustration of the magnetic hyperthermia therapy device for in vivo study of the nanocomplex efficiency on a U-87 MG-bearing nude mouse. (**E**) Tumor temperature monitoring over 2 cycles of MHT after a single injection of the nanocomplex. (**F**) Tumor volume (V/Vinital) versus time after treatment for single and combined therapy, with the arrows indicating the day of MHT application. Adapted from [42] with permission from Elsevier, 2017.

An alternative to chemotherapeutic drugs was proposed by Zhang et al. [42], who developed a biodegradable and thermosensitive magnetogel based on positively charged tumor necrosis factor-related apoptosis-inducing ligand (TRAIL) and hydrophobic superparamagnetic iron oxide nanoparticles, complexed with negatively charged poly(organophosphazene) polymers via ionic and hydrophobic interaction, respectively. TRAIL is a TNF cytokine family member and homotrimeric type 2 transmembrane protein-ligand, which specificity towards death receptors DR4 and DR5 overexpressed in cancer cells will selectively induce apoptosis. In addition, the combination with hyperthermia enhances TRAIL effects due to caspase activation release of cytochrome c and the aggregation or degradation of cellular FLICE-inhibitory protein [42]. Thus, the authors explored the synergy between multiple hyperthermia and TRAIL, which restored the sensitivity of intrinsic TRAIL-resistant U-87 MG cancer cells and enhanced TRAIL-induced apoptosis through the activation of caspase-3 and -8. Above all, the system demonstrated good in vivo results, further supporting the relevant use of protein-based therapeutics (Figure 6) [42].

Another stimulating advance observed in the field of bionanosystems was the development of lipogels by López-Noriega et al. [85]. The lipogels are based on chitosan/β-glycerophosphate hydrogel and lysolipid thermally sensitive liposomes (LTSLs), with a transition temperature of 41 °C, above which openings in the liposome are induced, promoting the release of the drug payload. Although not containing magnetic nanoparticles, the liposomes can be thermally activated by radiofrequency, microwaves or high-intensity-focused ultrasounds. This nanosystem combines the diffusion of free drug from the gel network and the limited release from encapsulated liposomes. Further, the liposomes were prepared by pH gradient method allowing a pH-sensitive release when the liposomes are subjected to an acidic pH, such as in the endosomal compartments [85]. On account of the promising properties of lipogels, the combination with magnetic nanoparticles might confer further potentialities to the magnetolipogels, due to the possibility of magnetically targeting towards a specific location and observation of the therapeutic efficiency by magnetic resonance imaging.

**Figure 7:**
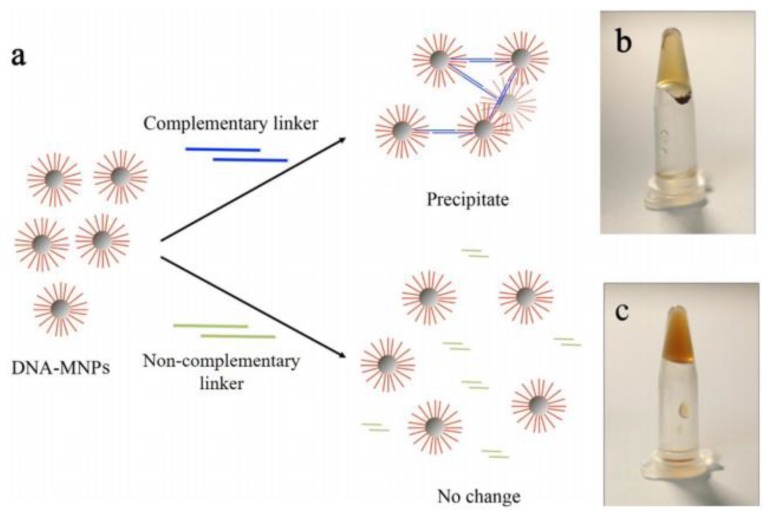
(**a**) Schematic representation of the assembly of DNA linkers to the DNA functionalized magnetic nanoparticles, where (**b**) the complementary linkers produced a precipitant in 10 min, while (**c**) the non-complementary linkers did not result in any change. Reprinted from [40] with permission from American Chemical Society, 2017.

Apart from drug delivery and hyperthermia, magnetogels have assisted a recent entry in the field of gene therapy. A pioneer work in this recent field was carried out by Ma and co-workers [40], who developed a DNA magnetogel with magnetic nanoparticles modified with DNA, being introduced in the DNA hydrogel matrix through hybridization (Figure 7). A remarkable characteristic is the possibility of the magnetogel adhering pure DNA hydrogel, which results in a single piece capable of being dragged under an applied magnetic field [40]. Undoubtedly, this work opens the way to future advancements in gene therapy, considering that such concept will allow the specific localization of the nanosystem in the desired target, the control over the DNA release and high quantity of transportable DNA, which combined with the possibility of drug delivery, may lead to a nanosystem of high potential for surpassing many of the already existing systems.

## 4. Magnetic Resonance Imaging

Magnetic resonance imaging (MRI) has been a target of intensive research for improved tumor imaging in the result of its high anatomical resolution with unlimited tissue depth and soft tissue contrast, taking into consideration the need to overcome the sensitivity and lack of specificity issues commonly associated with the conventional contrast agents [86]. For that purpose, bionanosystems have revolutionized MRI by exploring the enhanced permeation and retention (EPR) effect to preferentially passively accumulate in the tumor areas, but also by the possibility to conceptualize and tailor the physicochemical properties of the bionanosystem to actively direct it to the desired area [86]. Magnetic nanoparticles, mainly iron oxide nanoparticles, have been playing an important role as T_2_ contrast agents, due to their chemical stability and biodegradability, which can be explored in the monitorization of diseases and detection of injuries or deficiencies [87,88]. Though, it has also been reported that SPIONS suffer from peroxidase-like activity [56,57], which may eventually produce undesirable effects in the long-term due to the production of ROS, thus requiring a proper coating or the development of alternative nanoparticles. For example, cobalt nanoparticles exhibit a higher room temperature saturation magnetization (1422 emu cm^−3^) than iron oxide nanoparticles (395 emu cm^−3^), which can be explored to attain smaller particle core sizes without compromising sensitivity and achieve better magnetic resonance imaging contrast [87]. Nevertheless, such particles are easily prone to oxidation, thus requiring to be covered by an appropriate shell, to prevent the formation of Co^2+^, apart from being of difficult synthesis. These facts lead us back to the starting point where alternatives are required to the conventional iron oxide nanoparticles, but easily synthesized and with improved biocompatibility, which can be achieved by doping with transition metals such as Mn, Co, Ni and Zn, the MnFe_2_O_4_ nanoparticles showing the highest mass magnetization value (110 emu g^−1^), magnetic susceptibility and relativity values [87]. Nevertheless, improvements are needed, and it is in this field that magnetogels appear as a promising solution to the low sensitivity, which requires an understanding of the proton spin dephasing (T_2_ relaxation) mechanisms produced by the magnetic nanoparticles. These mechanisms are classified according to particle size, in which protons can diphase through a motional averaging regime (MAR), visit-limited regime (VLR) or static dephasing regime (SDR) [88]. When the distance travelled by the proton is longer than the distance between particles, full proton dephasing is not achieved in a single encounter, subjecting the proton to different magnetic environment along its travel, which might be refocused if an opposite polarity is found, thus retarding the overall relaxation rate [88]. Such is the motional averaging regime, where the relaxation rates are proportional to the particle size. However, as the particle size increases until a critical size, the proton enters in the visit-limited regime. In this mechanism, a full dephasing zone around the nanoparticle is defined where inside of it full dephasing is achieved while out of it only a minimal occurs, i.e., the process is dependent on the distance to the particle [88]. Thereby, as in this regime the protons spend enough time near one particle, it can be fully dephased in a single encounter. Finally, as the particles become large enough, the inter-particle distance gets longer than the distance travelled by the proton, subjecting the proton to an almost constant magnetic environment, which is defined as the static dephasing regime where an almost continuous dephasing is produced [88]. Naturally, these mechanisms have different implications for different bionanosystems based on magnetic nanoparticles. For example, in magnetoliposomes, protons are hindered from the dephasing zone near the particle surface by the hydrophobic coating, and so, in the MAR and VLR regimes the relaxation rates are both reduced [89]. On the contrary, magnetogels will have different mechanisms involved, as the hydrogel network will slow down water self-diffusing, which results in a longer proton retention time close to the particle surface and a decrease in the exchange of protons between the inner and outer coating environment [89]. Thus, a longer retention time will enhance MAR relaxation rate by increasing the dephasing at each encounter, but it will also reduce relaxation rates for the particles in the visit-limited regime [89].

**Figure 8:**
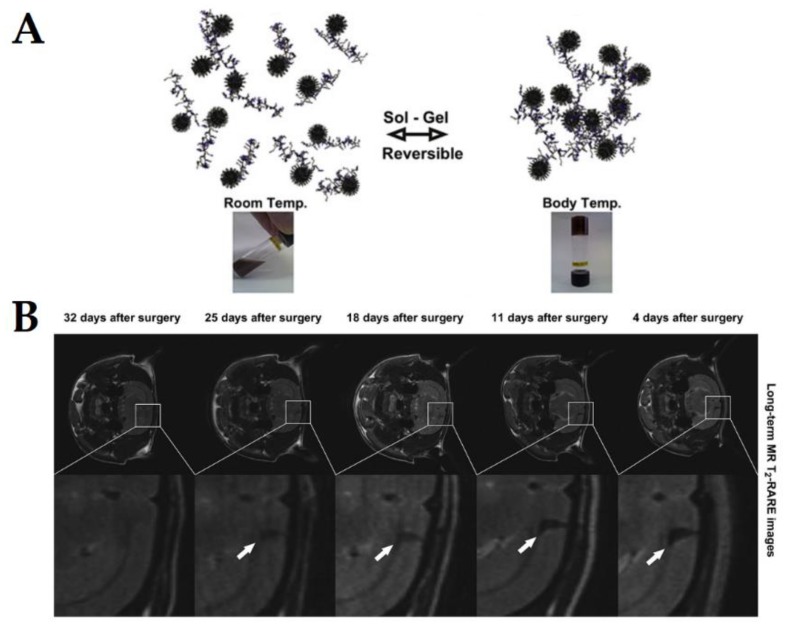
(**A**) Schematic representation of the thermosensitive responsive behavior of the poly(organophosphazene) and cobalt ferrite nanoparticles based magnetogel; (**B**) Time evolution of the in vivo degradation of the magnetogel for the assessment as a long-term MR contrast agent, having been injected into the righthand side of the frontal cortex region of the rat brain near the corpus callosum. Reprinted from [41] with permission from Elsevier, 2012.

Such properties have been explored for different applications, such as molecular or cellular detection, monitoring and tracking of cells in vivo [37]. For example, Paquet et al. [37] were able to enhance the transverse relaxation rate by up to 85% using SPIONS and a hydrogel made of acrylic acid, *N*,*N*′-methylene-bis-acrylamide and *N*-isopropylacrylamide. The authors showed that the increased hydrophilicity of the particle coating enhances interactions between protons and the magnetic core, which further increases the relaxivity [37]. This property combined with the high magnetic moment and the pH responsive hydrogel behaviour promotes pH-dependent and higher T_2_ relaxivities [37]. Thus, magnetogels are highly promising for magnetic resonance imaging applications as a result of the wide diversity of strategies possible of being used to enhance transverse relaxation rates. Moreover, magnetogels have been showing promising results in various in vivo assays, due to their huge structural and chemical flexibility. For example, Kim et al. [41] developed a thermosensitive magnetogel based on poly(organophosphazene) and cobalt ferrite nanoparticles coated with organic surfactants (oleic acid and oleylamine), which allowed non-covalent interactions between the L-isoleucine ethyl esters of the polymer and the nanoparticles hydrophobic coating during gelation (Figure 8A). Such nanosystem was estimated to be applicable as a long-term magnetic resonance contrast agent over 4–5 weeks after surgery, without blurring or spreading of the magnetogel and requiring a 4-day stabilization period [41]. Moreover, the authors reported that 4 days after injection using stereotactic surgery in rat brains, the magnetogel was well localized in the frontal cortex region, showing a strong negative MR T_2_ contrast (Figure 8B) [41]. These results, combined with properties such as the low cytotoxicity, thermosensitivity, biodegradability, biocompatibility and injectability, propels this nanosystem as highly promising for theranostics, when combined with therapeutic drugs, further supporting the potentiality of magnetogels [41].

**Figure 9:**
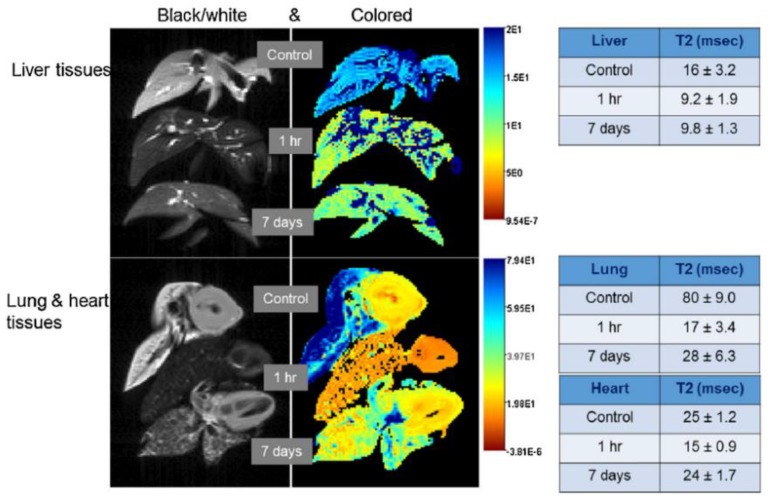
Magnetic resonance imaging of swiss mice model liver, lung and heart tissues after magnetogel injection via tail vein to monitor the in vivo biodistribution, the animals having been sacrificed at 1 h and 7 days after injection. Adapted from [38] with permission from Elsevier, 2015.

In another example, Chet et al. [90] developed a cellulose nanocrystal/silk fibroin-blended magnetogel containing ultrasmall superparamagnetic iron oxide nanoparticles that were used to non-invasively monitor magnetogel degradation and evaluate cartilage restoration through MRI. The authors reported that magnetogels and hydrogels loaded with bone-marrow mesenchymal stem cells took 8 weeks to show a marginal hyperintense signal due to occurrence of neocartilage into the defects alongside normal articular cartilage, only taking other 4 weeks until a continuous and smooth articular cartilage was formed [90]. Furthermore, it is possible to follow up the biodistribution of magnetogels through MRI and achieve controlled drug release. Jaiswal et al. [38] developed a magnetogel based on poly(*N*-isopropylacrylamide) and Fe_3_O_4_ nanoparticles functionalized with polyethylene glycol (PEG), which was loaded with doxorubicin and incubated with bladder cancer T-24 cell lines, to evaluate the effect of an external radiofrequency field and applied in swiss mice model via tail vein to monitor magnetogel accumulation. The authors reported that one-hour application of a 375 Oe radiofrequency field resulted in 40% cells under apoptotic condition 24 h after treatment, compared to only 13% without radiofrequency field exposure, attaining a 95% cell death due to synergy between the heat generated by the RF field and doxorubicin release [38]. In addition, the magnetogel was found to preferentially accumulate in lungs 1 h after administration, with the heart showing a similar trend and liver showing no contrast enhancement for 7 days (Figure 9) [38].

Although these works demonstrate the potentiality of magnetogels for theranostics, improvements on the signal-to-noise ratio are always required and also the avoidance of repeated administration of contrast agents, without affecting accessibility and biodegradability of the developed system. Thus, the development of magnetogels based on chelating hydrogelators might provide a powerful nanosystem for MRI, taking into consideration the optimal results attained by hydrogelators alone. For example, Chan et al. [86] developed a self-assembled cholesterol and acryloyl modified pullulan hydrogelator containing gadolinium-chelating cross-linkers (1,4,7,10-tetraazacyclo-dodecane-1,4,7,10-tetraacetic acid (DOTA)) to confer magnetic properties and stabilize the material. These authors reported a 4 h high-level accumulation in 4T1 tumors in mice after injection and a high T_1_ signal enhancement that lasts up to 7 days, without damage or toxicity in major organs up to 3 months [86].

## 5. Future Perspectives, Goals and Conclusions

Magnetogels have been showing promising and interesting results in various fields from biosensing to theranostics. Nevertheless, instead of being restrained to superparamagnetic ferrite nanoparticles, the combination of magnetic and plasmonic effects should be a future goal, for example by the development of core/shell nanoparticles (e.g., with a magnetic core and a gold shell) or the development of magnetic nanoparticles decorated with plasmonic gold particles. Indeed, gold has been a remarkable material, from Chinese traditional medicine to modern therapeutic applications, such as the use of gold salts for the treatment of rheumatoid arthritis [91]. Apart from that, the unique optical and physical properties, combined with the possibility to confer multiple surface functionalities, has promoted the use of gold nanoparticles in several applications [91,92]. Besides some aspects in common with other nanoparticles, such as the size and shape dependence of the optoelectronic properties and the great surface-to-volume ratio, gold furnishes nanoparticles with excellent biocompatibility, low toxicity and the possibility of exploring the surface plasmon resonance phenomenon and quenching of fluorescence. The surface plasmon resonance (SPR) consists in the conductive electrons collective motion in relation to the crystal lattice, due to an externally applied electric field, which produces a depolarizing electric field acting as a restoring force and behaves as a damped oscillator due to the interactions between electrons and the environment/vicinity [93]. Nevertheless, for particle sizes smaller than 2 nm, the quantization energy of the electronic levels becomes high enough to the point that the collective oscillation is hindered. Therefore, as the surface plasmon resonance is highly sensible to the particle size and the environment (particularly, the dielectric constant), it might function as a sensor to the presence of molecules bonded or in the proximity of the nanoparticle surface [93]. For example, the presence of electron donors (e.g., alkanethiols or alkylamines) induces a bathochromic shift, while acceptors (e.g., phosphines) induce a hypsochromic shift of the plasmon frequency. In addition, the plasmon band is sensible to the core charge, temperature, shape and proximity of other particles, i.e., the particle aggregation induces interparticle plasmonic coupling, which results in a bathochromic shift of the plasmon frequency, bandwidth widening and a solution color change from red to blue [92]. Moreover, gold nanoparticles strongly quench fluorescence through Förster resonance energy transfer (FRET), due to the superposition of the emission spectra of fluorophores and the absorption band of nanoparticles surface plasmon. Though, quenching can also be a result of photoinduced electron transfer modulated by the charging or discharging of the gold nucleus, thus becoming useful for the development of sensors [92]. These properties, together with the diversity of surface functionalities, have contributed to the application of gold nanoparticles in different areas of technological interest such as biosensors, clinic chemistry methods, immunologic assays, photothermolysis of tumor cells, detection and control of microorganisms, transport of drugs and monitorization of biological cells [91]. In the field of bionanosystems, Rittikulsittichai et al. [94] made a pioneer work by developing magnetogel particles based on the thermoresponsive poly(NIPAM-co-AA), silica-coated Fe_3_O_4_ nanoparticles and gold nanorods, which is responsive to several stimuli such as temperature, pH, light and an applied external magnetic field. The authors demonstrated that upon a stimulus, the magnetogel could undergo a systematic and reversible variation in the hydrodynamic diameter, due to swelling and deswelling of the hydrogel network, which affects the gold nanorods optical properties. Moreover, the application of a 300 mW at 808 ± 10 nm electromagnetic radiation induced a temperature change from 22 °C to 36 °C in 5 min, while an alternating magnetic field of 10 kA/m at 100 kHz changed temperature from 34 °C to 40 °C in 10 min [94]. Such work demonstrates the promising synergy between magnetic and plasmonic effects to remotely control drug delivery and enhance hyperthermia therapy.

Magnetogels could also be used as a nanosystem for gene delivery and therapy, in combination with drug delivery, magnetic and plasmonic hyperthermia. For example, Eguchi et al. [95] developed a DNA-based hydrogel cross-linked through Au-S interactions to gold nanoparticles, where the DNA acts as the network polymer and the nanoparticles as nucleation, overcoming the possible leaching out of nanoparticles. The authors demonstrated that the DNA required for hydrogelation is long enough to encode proteins, this being evaluated for GFP (6012 bp) by incubating with cell lysates of *E. coli*, attaining 0.6 μg in 50 mL in one hour [95]. Another interesting aspect is that gold nanoparticles have been shown remarkable results for tissue regeneration, mainly by stimulation of osteogenic differentiation associated with the inhibition of the receptor activator of nuclear factor-κb ligand pathway towards osteoclast formation in bone marrow-derived macrophages and activation of the p38 mitogen-activated protein kinase (MAPK) pathway, after intracellular uptake by mesenchymal stem cells [96]. Heo et al. [96] developed a methacrylated gelatin-based hydrogel containing gold nanoparticles and showed that higher nanoparticle content increased proliferation rate and osteogenic differentiation of human adipose-derived stem cells, apart from stimulating bone regeneration of parietal bone defects. Furthermore, magnetogels could be combined with magnetoliposomes to improve control over drug diffusion, increase the diversity of drugs passible of being incorporated, enhance MRI contrast by introducing clusters of nanoparticles in a medium containing magnetic nanoparticles (that might enhance the regimes previously described), and add enzymatic and genetic therapy features.

Magnetogels have exhibited many advantages when compared to the standalone components, which have promoted the entrance into a wide diversity of applications, from biosensors to contrast agents. Nevertheless, such nanosystem is very recent and there are still few works which explore their promising properties in hyperthermia and MRI combined with drug delivery. Moreover, most of the published studies are focused on the use of iron oxide nanoparticles and not on other types of nanoparticles with advantageous properties, such as transition metal doped ferrites or cobalt nanoparticles. As has been discussed, gold exhibits interesting properties such as the surface plasmon resonance, affinity towards sulphur and cell differentiation stimulation that should be readily explored for the combined application of photothermia and magnetic hyperthermia to synergistically enhance potent anticancer drug effects on the desired target. Interestingly, the use of superparamagnetic property of gold nanoparticles when in the presence of sulphur or the easy binding of ligands and polymers [91,92,93], allows the development of magnetogels solely based on gold nanoparticles and multifunctional polymers, thus attaining nanosystems that can work as anti-inflammatory, anti-pathogenic and regenerative agents on the treatment of wounds and profound tissue defects.

Apart from gold, silver might also be of interest due to its anti-pathogenic activity, as it may help to reduce the need of developing every year new antibiotics, to overcome the astonishing rising population of resistant bacteria, which is a path that will inevitably end in higher susceptibility to untreatable pathogenicities.

Nevertheless, apart from introducing plasmonic properties in the magnetogels to attain more control over their behavior and widening, and even more, their applications, other magnetic nanoparticles should be readily explored in the development of magnetogels for biomedical applications, such as the CoNi alloys for magnetic resonance imaging [97]. Although, such nanomaterial may require a proper coating to confer adequate chemical stability and biocompatibility for biomedical applications. A major advantage of magnetogels is the reduction of nanoparticles toxicity, as shown by Finetti et al. [98], who developed a magnetogel based on carboxymethylcellulose and CoFe_2_O_4_ nanoparticles coated with (3-aminopropyl)trimethoxysilane acting as cross-linkers. The authors demonstrated that the endothelial permeability, oxidative stress, cytoskeleton organization and inflammatory markers associated with endothelial toxicity remained unchanged in magnetogels, while for the nanoparticles an increase was observed. Therefore, such toxicity reduction effect could be used to develop and explore other magnetogels comprising CoFe_2_O_4_ nanoparticles or other nanomaterials that have not yet been utilized due to such a problem. However, the MnFe_2_O_4_ nanoparticles are recommendable from all the transition metal doped-ferrites owing to their biocompatibility, strong T_2_ phase contrast for magnetic resonance imaging, and higher magnetic susceptibility when compared with other ferrite nanoparticles [99]. An example of their potential for hyperthermia cancer therapy is a chitosan-based magnetogel developed by Kim et al. [99], which showed a remarkable specific absorption rate (SAR) of 1.45 W/g at 266 kHz and 653 Oe.

However, besides the necessity to explore other magnetic nanoparticles to improve hyperthermia, MRI contrast and add photothermia properties, magnetogels should be focused on the use of biodegradable and biocompatible hydrogelators, such as polysaccharides, DNA or peptide-based hydrogelators. Such materials should be low molecular weight hydrogelators to reduce their impact over the magnetic response of the magnetic nanoparticles to an externally applied magnetic field. Also, further functional properties could be added such as gadolinium chelators, moieties to cross-link hydrogelators and nanoparticles (to reduce the possibility of leaching out from the hydrogel network) and add functionalities to passively direct hydrogels to a specific target. For example, the use of the naproxyl group in a hydrogelator molecular structure will afford selectivity towards cyclooxygenase-2 [13]. The hydrogelators should also be responsive to various stimuli, so to adapt the magnetogel to different situations or to attain different MRI contrasts.

Therefore, magnetogels appear as strong candidates for future personalized therapies and theranostic applications, which are potentiated by the strong fertile ground of functionalities and properties passible of being developed, optimized and adapted for the different applications, whether it is tissue engineering, drug delivery, hyperthermia, MRI or all together in the same nanosystem.

## Figures and Tables

**Table 1:** Examples of biomedical applications of magnetogels according to the utilized nanoparticles and hydrogelators.

Nanoparticle	Hydrogelator	Application	Reference
Iron oxide	Alginate-*g*-heparin	Cell differentiation	[33]
	PEGMMA and PEGDMA	Cancer therapy	[34]
	Poly(*N*-isopropylacrylamide)	Cancer therapy	[35,36]
		MRI	[37,38]
	Chitosan	Cancer therapy	[39]
	DNA	Gene therapy	[40]
	Poly(organophosphazene)	Cancer therapy	[41]
Cobalt ferrite		MRI	[42]
